# l‐Homoarginine supplementation prevents diabetic kidney damage

**DOI:** 10.14814/phy2.14235

**Published:** 2019-09-25

**Authors:** Michael D. Wetzel, Ting Gao, Manjeri Venkatachalam, Sidney M. Morris, Alaa S. Awad

**Affiliations:** ^1^ Department of Medicine University of Texas Health Science Center at San Antonio San Antonio Texas; ^2^ Department of Medicine Penn State University College of Medicine Hershey Pennsylvania; ^3^ Department of Pathology University of Texas Health Science Center at San Antonio San Antonio Texas; ^4^ Department of Microbiology & Molecular Genetics University of Pittsburgh Pittsburgh Pennsylvania

**Keywords:** Diabetic nephropathy, l‐homoarginine, nitric oxide

## Abstract

l‐homoarginine is an endogenous, non‐proteinogenic amino acid that has emerged as a new player in health and disease. Specifically, low l‐homoarginine levels are associated with cardiovascular diseases, stroke, and reduced kidney function. However, the role of l‐homoarginine in the pathogenesis of diabetic nephropathy (DN) is not known. Experiments were conducted in 6‐week‐old *Ins2^Akita^* mice supplemented with l‐homoarginine via drinking water or mini osmotic pump for 12 weeks. Both plasma and kidney l‐homoarginine levels were significantly reduced in diabetic mice compared to nondiabetic controls. Untreated *Ins2^Akita^* mice showed significant increases in urinary albumin excretion, histological changes, glomerular macrophage recruitment, the inflammatory cytokine KC‐GRO/CXCL1, and urinary thiobarbituric acid reactive substances (TBARS) excretion as an indicator of oxidative stress, along with a significant reduction in kidney nitrate + nitrite levels compared to control mice at 18 weeks of age. In contrast, l‐homoarginine supplementation for 12 weeks in *Ins2^Akita^* mice, via either drinking water or mini osmotic pump, significantly reduced albuminuria, renal histological changes, glomerular macrophage recruitment, KC‐GRO/CXCL1 levels, urinary TBARS excretion, and largely restored kidney nitrate + nitrite levels. These data demonstrate that l‐homoarginine supplementation attenuates specific features of DN in mice and could be a potential new therapeutic tool for treating diabetic patients.

## Introduction

In the United States, diabetes is the major cause of end‐stage renal disease (ESRD), accounting for more than 40% of ESRD patients, making it a heavy burden for the health care system (Boyle et al. 2010). Early histological changes in diabetic nephropathy (DN) include glomerular hyperfiltration, glomerular hypertrophy, followed by glomerular basement membrane thickening, endothelium dysfunction, and mesangial matrix accumulation. As the disease progresses, the urinary albumin excretion rate (UAER) increases, which leads to glomerular sclerosis and ultimately ESRD.

l‐homoarginine is an endogenous amino acid in many species that is not involved in protein synthesis (Pilz et al. 2015). l‐homoarginine is synthesized by mitochondrial arginine:glycine amidinotransferase (AGAT) in kidneys using l‐arginine and l‐lysine as substrates (Ryan and Wells 1964; Choe et al. 2013) and is distinguished from l‐arginine by an additional methylene group in its structure (Pilz et al. 2015).

Plasma concentrations of homoarginine are inversely correlated with the risk of cardiovascular disease and overall mortality (Marz et al. 2010; Jud et al. 2018), increased risk for fatal strokes (Haghikia et al. 2017), congestive heart failure and left ventricular hypertrophy (Atzler et al. 2013, 2017, Pilz et al. 2014a; Bahls et al. 2018), aging (Marz et al. 2010; Atzler et al. 2014b), smoking (Zwan et al. 2013; Sobczak et al. 2014; Atzler et al. 2014a; Vogl et al. 2015), body mass index (Marz et al. 2010; Atzler et al. 2014a; Pilz et al. 2014b), and pregnancy (Valtonen et al. 2008). In addition, the effects of homoarginine on nitric oxide (NO) synthesis have been linked with increased risk of stroke and atherosclerosis as a reduction in homoarginine levels increased the aortic intima‐media thickness (Haghikia et al. 2017). Low plasma homoarginine concentration is also significantly associated with the progression of chronic kidney disease (Drechsler et al. 2013) and progression to dialysis and mortality (Ravani et al. 2013), indicating that low plasma homoarginine concentrations might be an early indicator of kidney failure and a potential target for the prevention of disease progression. However, the role of homoarginine in the pathogenesis of DN is not known.

These findings suggested that changes in l‐homoarginine levels or metabolism may be involved in chronic renal injury. However, there is little information regarding the possible role of l‐homoarginine in diabetic renal injury.

Therefore, we investigated whether changes in l‐homoarginine levels play a role in DN. Using diabetic *Ins2^Akita^* mice as a model of type‐1 diabetes, this current study demonstrates that plasma and kidney homoarginine levels were reduced in diabetic mice compared to nondiabetic control mice. l‐homoarginine supplementation significantly reduced kidney injury, as evidenced by reduced albuminuria, histologic kidney damage, glomerular macrophage recruitment, inflammatory cytokines, urinary thiobarbituric acid reactive substances (TBARS) excretion, along with significant increases in kidney nitrate + nitrite levels. Taken together, our data suggest that l‐homoarginine supplementation might be a promising therapeutic modality for DN.

## Materials and Methods

### Animal model

All animal experiments were performed in *Ins2^Akita^* and their wild‐type littermates (DBA background; Jackson Laboratories, Bar Harbor, ME). The experiments started at 6 weeks of age. As recommended by the Animal Models of Diabetes Complication Consortium as an optimal model of DN, *Ins2^Akita^* mice develop hyperglycemia at 3–4 weeks of age (Breyer et al. 2005; Brosius et al. 2009). All the mice were euthanized at 18 weeks of age. Mouse plasma and 24‐hour urine were collected, and kidneys were removed for further studies. All the animal experiments were approved by Pennsylvania State University College of Medicine Institutional Animal Care and Use Committee.

### l‐homoarginine administration

*Ins2^Akita^* mice at 6 weeks of age were supplemented with l‐homoarginine (Sigma, St. Louis MO, Cat #: H1007) for 12 weeks either in drinking water at a concentration of 50 mg/L or by continuous subcutaneous infusion via a mini osmotic pump (Alzet, Durect, Palo Alto, CA) at a dose of 0.72 mg/kg/day (Pump was changed after 6 weeks). All mice had free access to drinking water.

### Immunohistochemistry

Mouse kidney tissues were fixed in 10% formalin and embedded in paraffin, and 3‐*μ*m sections were cut. Immunohistochemistry was performed on paraffin‐embedded sections with anti‐mouse Mac‐2 antibody (clone M3/38; Cedarlane, Burlington, NC) as previously described (You et al. 2013a, 2014). Twenty glomeruli were examined at 40× in a blinded manner. Images were taken with an Olympus BX51 microscope and DP71 digital camera using Microsuite Basic 2.6 image software. Images were obtained with 100× (oil) objective with a total magnification of 1000×.

### Renal histopathology

Kidneys were fixed in 4% paraformaldehyde, embedded in paraffin, and 5‐*μ*m sections were cut. Sections were stained with periodic acid–Schiff (PAS) stain, all glomeruli were examined at 400× in a blinded manner, and scores were averaged. All images were obtained with a Nikon Eclipse E600 microscope and Nikon DXM1200 camera. Images were taken at 400x magnification.

### Analytical methodology

Blood glucose was measured using Accu‐Chek glucometer and urine albumin concentration was measured by ELISA using an Albuwell M kit (Exocell, Philadelphia, PA) as described previously (Awad et al. 2011, 2015b; Morris et al. 2017). Urinary TBARS Assay was performed according to the instruction provided by Models of Diabetic Complication Consortium (AMDCC) as described previously (You et al. 2013b, 2014). Plasma and kidney arginine levels were determined by liquid chromatography–mass spectroscopy and amino acid assays as previously described (You et al. 2014).

### Homoarginine assay

Mouse kidneys were homogenized in 300‐*μ*L 3M perchloric acid (Fisher Scientific, Fair Lawn, NJ) per 10 mg of tissue. After centrifugation at 4°C, the supernatants were used for amino acid assay as previously described (You et al. 2014).

### Kidney nitrate and nitrite assay

Kidney lysates were homogenized in 1 × PBS, pH 7.4, and centrifuged at 4°C. Supernatants were transferred to a clean tube and protein concentration was determined by BCA protein assay. Nitrate and nitrite were measured using a Nitrate/Nitrite Colorimetric Assay Kit (Cayman Chemical, Ann Arbor, MI, cat #: 780001) according to manufacturer's instructions (You et al. 2014).

### MSD multi‐spot assay

Mouse KC‐GRO/CXCL1 was measured in 90 *µ*g of kidney lysate protein using a Multi‐spot assay system (Mouse proinflammatory panel 1, Meso Scale Diagnostics, Rockville, MD) performed as per manufacturer's instructions.

### Statistical analysis

Comparisons between groups were conducted using SPSS software (version 19.0, SPSS, Chicago, IL). Results are expressed as mean ± SEM. One‐way ANOVA was used to compare significance between more than two groups. A *P* value of <0.05 represented significant difference.

## Results

### Type‐1 diabetes reduces plasma and kidney l‐homoarginine levels

**Figure 1:**
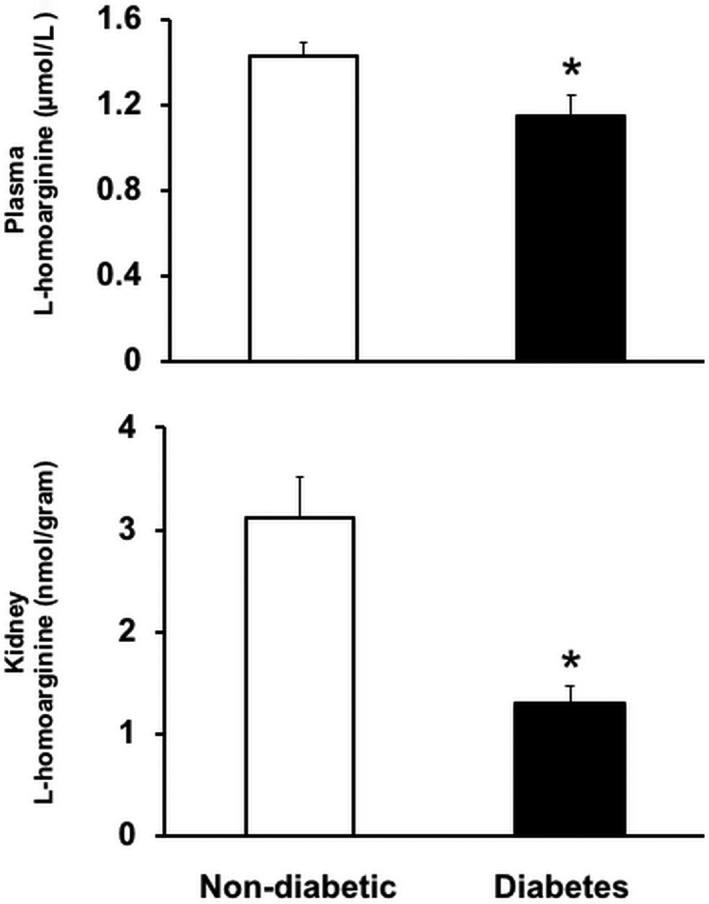
Plasma and kidney l‐homoarginine levels were reduced in type‐1 diabetic mice. Plasma and kidney samples from diabetic and nondiabetic mice at 18 weeks of age were used to measure l‐homoarginine levels using a liquid chromatography–mass spectrometry system. Results are presented as mean ± SEM. *P < 0.05 compared to nondiabetic mice.

Plasma samples from 18‐week‐old diabetic mice were analyzed for l‐homoarginine levels. Both plasma and kidney l‐homoarginine levels were significantly reduced in type‐1 diabetic mice compared to nondiabetic control mice (Fig. 1), indicating that diabetes alters circulating l‐homoarginine levels.

### l‐homoarginine supplementation did not alter gross characteristics of diabetic mice

l‐homoarginine supplementation via drinking water or osmotic pump had no effect on the increased blood glucose levels, increased water consumption, decreased body weight, or increased kidney weight/body weight ratio in diabetic mice (Table 1).

**Table 1:** Characteristics of mice.

	Nondiabetic	Diabetes	Diabetes + oral HA	Diabetes + pump HA
Number	10	9	9	7
BW (g)	33 ± 0.8	26 ± 0.5^a^	25 ± 0.7^a^	27 ± 0.8^b^
BG (mg/dL)	156 ± 11	501 ± 65^a^	542 ± 50^a^	638 ± 42^a^
WI (mL/24 h)	1.9 ± 0.4	6.8 ± 0.4^a^	5.6 ± 0.7^c^	7.5 ± 0.7^a^
KW/BW	0.96 ± 0.04	1.38 ± 0.08^c^	1.35 ± 0.08^c^	1.25 ± 0.06^b^

Diabetic mice were supplemented with homoarginine via drinking water or osmotic pumps for 12 weeks. HA, homoarginine; BW, Body weight; BG, Blood glucose; WI, Water intake; KW/BW, kidney weight/body weight.

a *P* < 0.0001.

b *P* < 0.001.

c *P* < 0.0005 versus nondiabetic.

### l‐homoarginine supplementation reduced albuminuria in diabetic mice

**Figure 2:**
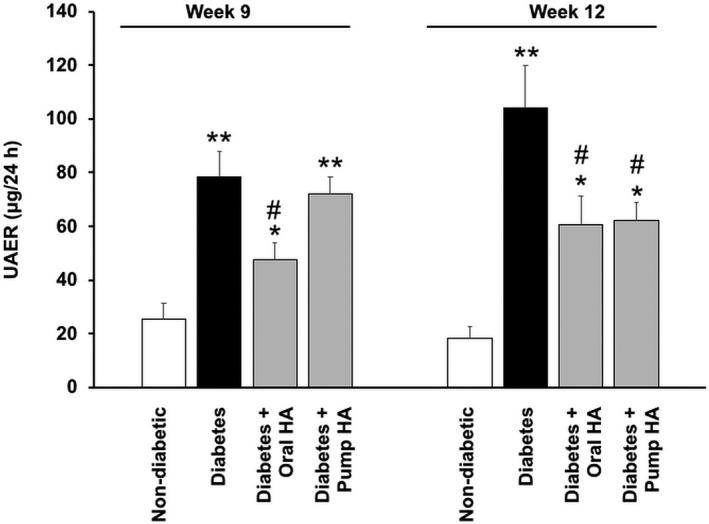
l‐homoarginine supplementation reduces urine albuminuria in diabetic mice. l‐homoarginine was supplemented to *Ins2^Akita^* mice for 12 weeks via either drinking water or mini osmotic pumps as indicated. Twenty‐four hour urine was collected for the measurement of UAER after 9 and 12 weeks of supplementation. Data are presented as mean ± SEM. *P < 0.05; **P < 0.01 compared to nondiabetic controls mice; ^#^ *P* < 0.05 compared to diabetic mice.

**Figure 3:**
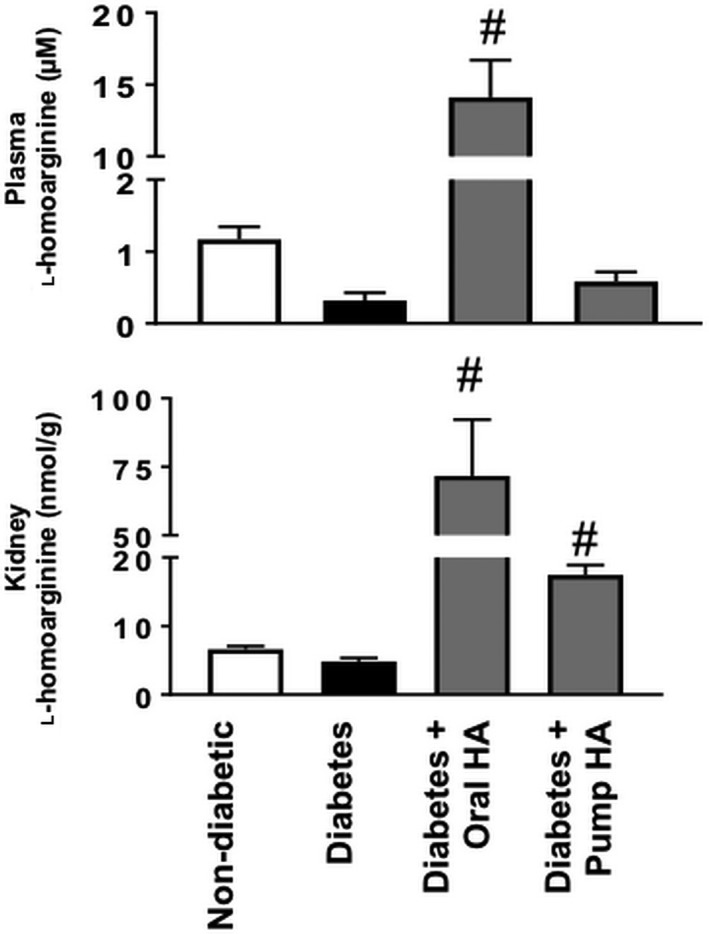
l‐homoarginine supplementation increased kidney homoarginine levels in diabetic mice. Plasma and kidney samples were used after 12 weeks of diabetes using a liquid chromatography–mass spectrometry system or ELISA assay. Results are presented as mean ± SEM. ^#^ *P* < 0.0001 compared to diabetic mice.

As shown in Figure 2, *Ins2^Akita^* mice displayed significantly higher UAER than nondiabetic controls. l‐homoarginine supplementation by either drinking water or osmotic pumps for 12 weeks in *Ins2^Akita^* mice significantly reduced UAER compared to untreated *Ins2^Akita^* mice. In addition, l‐homoarginine supplementation by drinking water in *Ins2^Akita^* mice significantly reduced UAER at week 9 compared to untreated *Ins2^Akita^* mice. Importantly, l‐homoarginine supplementation significantly increased kidney homoarginine levels (Fig. 3).

### l‐homoarginine supplementation decreased renal histological changes in diabetic mice

**Figure 4:**
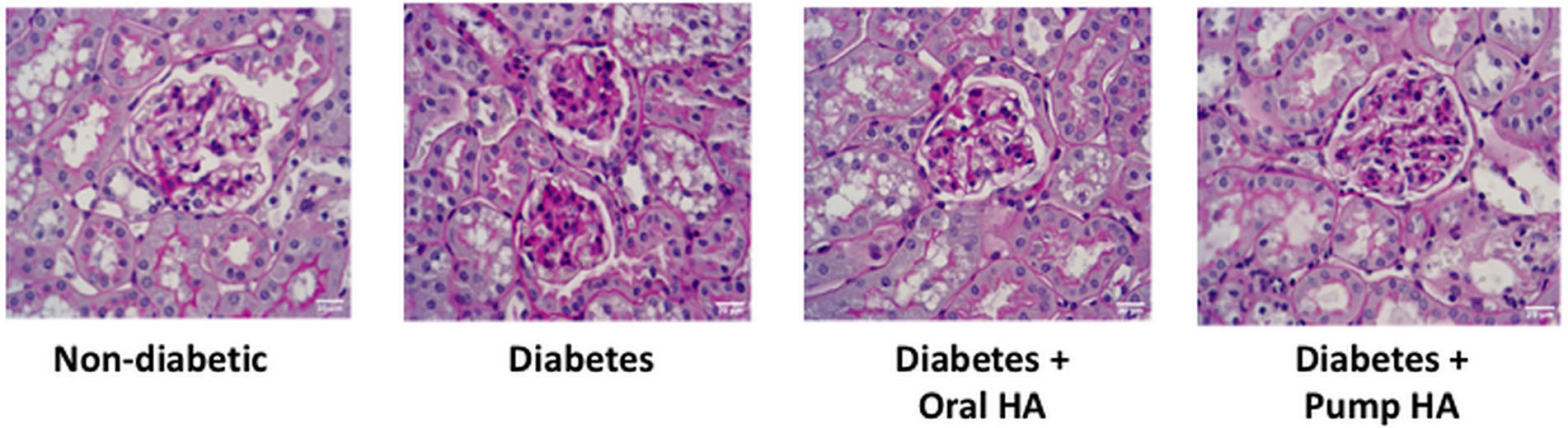
Effects of l‐homoarginine on kidney pathology. Paraffin‐embedded mouse kidney sections were subjected to periodic acid–Schiff (PAS) staining. Scale bar represents 20 *µ*m.

Periodic acid–Schiff staining showed significantly increased glomerular cellularity and mesangial expansion at 18 weeks of age in *Ins2^Akita^* mice versus nondiabetic controls (score of 1.5 vs. 0.25, respectively). l‐homoarginine supplementation by either drinking water or osmotic pumps for 12 weeks in *Ins2^Akita^* mice significantly reduced glomerular changes (score of 0.625 and 0.375, respectively) compared to untreated *Ins2^Akita^* mice (Fig. 4).

### l‐homoarginine supplementation reduces glomerular macrophage infiltration in diabetic mice

**Figure 5:**
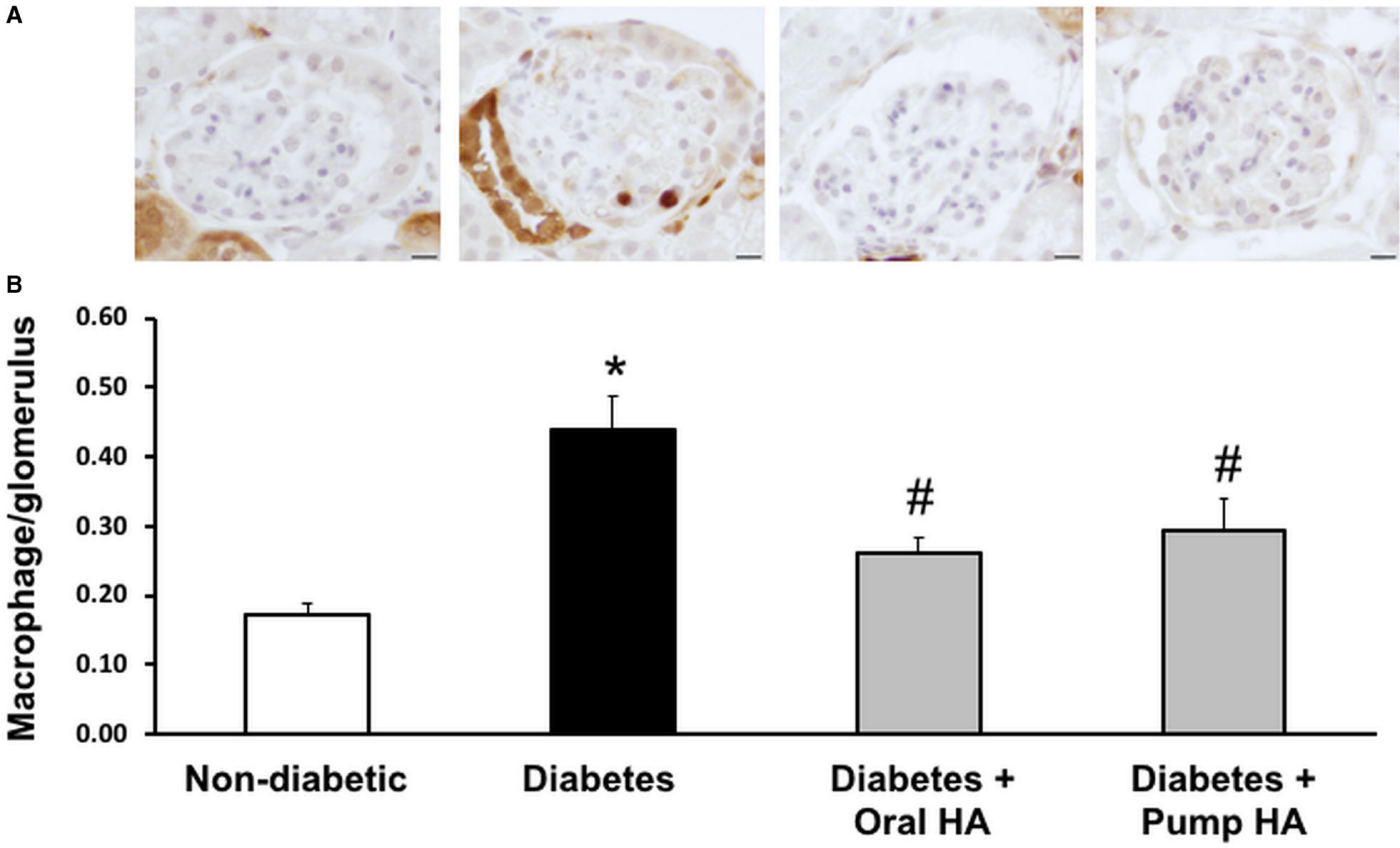
l‐homoarginine supplementation reduces glomerular macrophage infiltration in diabetic mice. (A) Glomerular macrophage recruitment was visualized by immunohistochemical staining using Mac‐2 antibody 12 weeks after l‐homoarginine supplementation. Macrophage numbers per glomerulus were counted in a blinded manner. Scale bar in images represents 10 *µ*m. (B) The number of glomerular macrophages was counted in 20 glomeruli per section (number of macrophages in glomeruli divided by the number of glomeruli) in blinded fashion under 40× magnification and averaged. Results are presented as mean ± SEM. *P < 0.01 compared to nondiabetic control mice; ^#^ *P* < 0.05 compared to diabetic mice. *N* = 10 for nondiabetic, 9 for diabetes and diabetes + oral HA, 7 for diabetes + pump HA.

Consistent with previous studies (You et al. 2013a; Awad et al. 2015a), Mac‐2 staining in untreated *Ins2^Akita^* mice showed significant increases in glomerular macrophages compared with untreated controls. l‐homoarginine supplementation by either drinking water or osmotic pumps for 12 weeks in *Ins2^Akita^* mice had significantly reduced glomerular macrophage recruitment compared to untreated *Ins2^Akita^* mice (Fig. 5).

### l‐homoarginine supplementation reduces kidney KC‐GRO (CXCL1) level in diabetic mice

**Figure 6:**
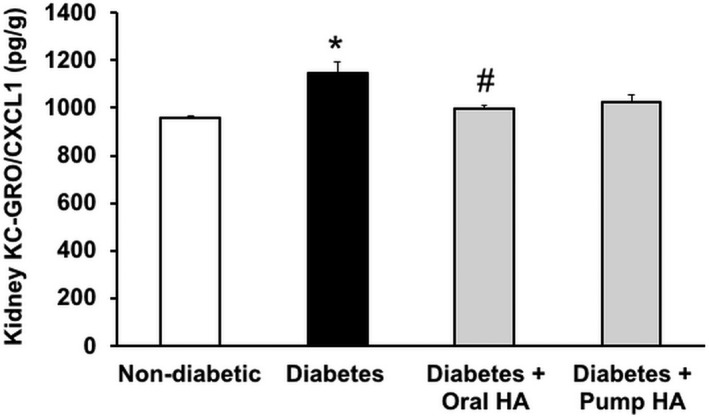
l‐homoarginine supplementation reduces kidney KC‐GRO/CXCL1 levels in diabetic mice. A MSD multi‐spot assay was used to measure kidney KC‐GRO levels. Data are presented as mean ± SEM. *P < 0.0005 compared to nondiabetic control mice; ^#^ *P* < 0.005 compared to diabetic mice.

We demonstrated previously that inflammation is a critical determinant in the pathophysiology of DN (Awad et al. 2011; You et al. 2017). KC‐GRO/CXCL1 is a cytokine that plays an important role in leukocyte recruitment, and mediates inflammation in diabetes (Citro et al. 2015). Kidney KC‐GRO/CXCL1 significantly increased in untreated *Ins2^Akita^* mice compared to nondiabetic controls, an effect that was attenuated by oral l‐homoarginine supplementation (Fig. 6).

### l‐homoarginine supplementation reduces TBARS, an indicator of oxidative stress in diabetic mice

**Figure 7:**
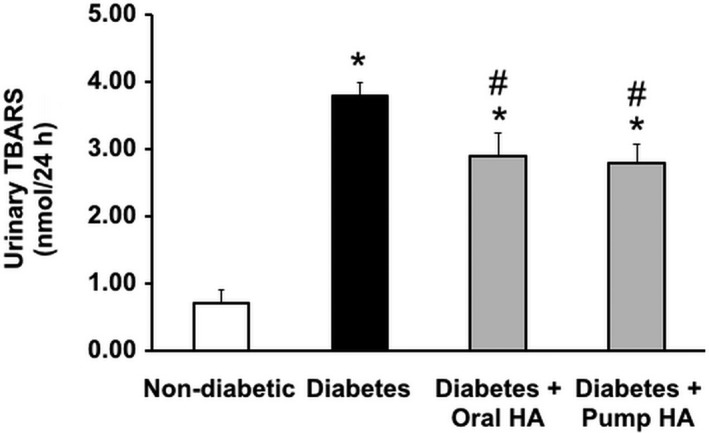
l‐homoarginine supplementation reduces urinary TBARS excretion in diabetic mice. Twenty‐four hour urine was collected at 18 weeks of age for the measurement of TBARS. Data are presented as mean ± SEM. *P < 0.0001 compared to nondiabetic controls mice; ^#^ *P* < 0.05 compared to diabetic mice.

Urinary TBARS levels were used as an indicator of oxidative stress as previously reported (You et al. 2013b, 2014). Whereas levels of urinary TBARS greatly increased in untreated *Ins2^Akita^* mice compared to nondiabetic controls; urinary TBARS excretion was significantly attenuated in diabetic mice that received l‐homoarginine supplementation compared to untreated *Ins2^Akita^* mice (Fig. 7).

### l‐homoarginine supplementation increases kidney nitrate and nitrite production in diabetic mice

**Figure 8:**
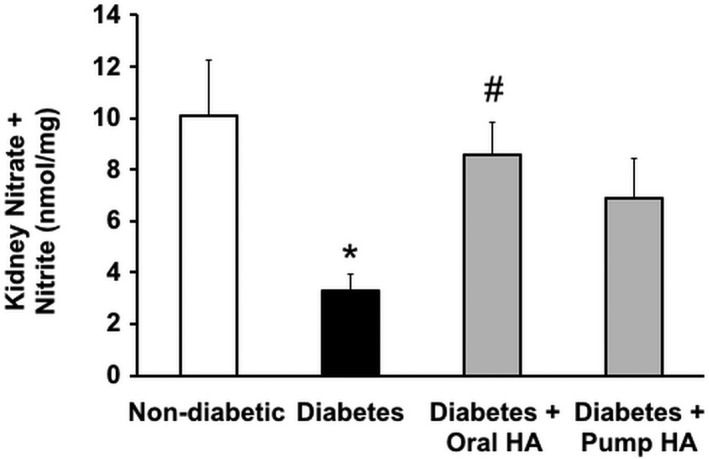
l‐homoarginine supplementation increases renal nitrate and nitrite concentration in diabetic mice. Mouse kidney lysates were used to measure nitrate and nitrite concentration after 12 weeks of l‐homoarginine supplementation. Data are presented as mean ± SEM. *P < 0.01 compared to nondiabetic control mice; ^#^ *P* < 0.05 compared to diabetic mice.

Nitric oxide plays a critical role in DN. Previous reports demonstrated that NO inhibition or deficiency exacerbates kidney dysfunction in diabetic mice (Zhao et al. 2006; Wang et al. 2011). Consistent with our previous report (You et al. 2015), kidney nitrate + nitrite levels were significantly reduced in *Ins2^Akita^* mice compared to nondiabetic controls. Oral l‐homoarginine supplementation significantly increased kidney nitrate + nitrite levels compared to untreated *Ins2^Akita^* mice (Fig. 8).

### l‐homoarginine supplementation did not affect arginine levels in diabetic mice

**Figure 9:**
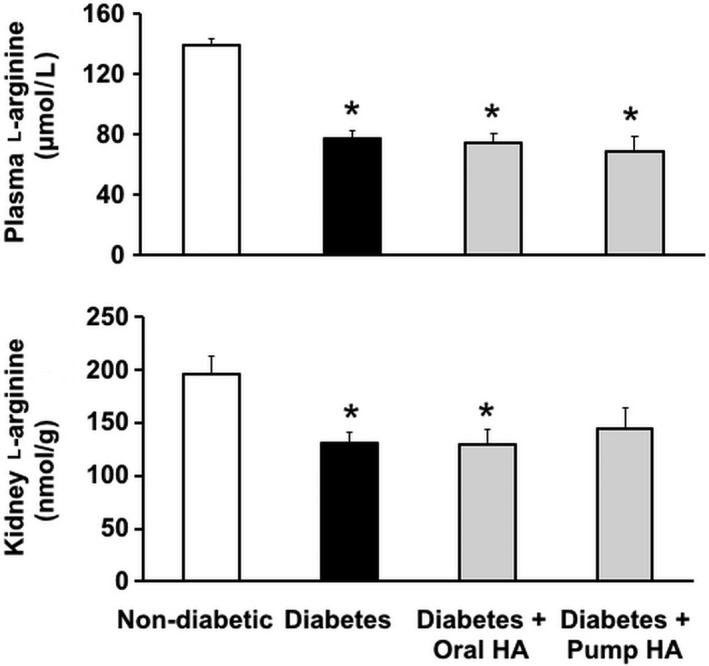
The effect of l‐homoarginine supplementation on l‐arginine levels. Mouse plasma and kidney tissues were prepared to measure l‐arginine levels using a liquid chromatography–mass spectrometry system. Data are mean ± SEM. *P < 0.01 compared to nondiabetic control mice.

As arginase‐2 inhibition or deletion ameliorates diabetic kidney injury (Morris et al. 2011; You et al. 2013b, 2014) and arginase inhibition prevents the reduction in kidney nitrate + nitrite levels in diabetic mice (You et al. 2015), we investigated whether homoarginine has any effect on l‐arginine levels in circulation and in the kidneys. Although plasma l‐arginine levels were shown to be reduced in streptozotocin‐induced diabetic DBA/2J mice, (You et al. 2014) plasma l‐arginine levels have not been reported previously for diabetic *Ins2^Akita^* mice. We therefore evaluated l‐arginine availability in *Ins2^Akita^* mice as indicated by levels of l‐arginine in plasma and kidney. As shown in Figure 9, diabetes significantly reduced the levels of l‐arginine in both plasma and kidney, but l‐homoarginine supplementation did not restore l‐arginine levels to nondiabetic levels in either plasma or kidney.

## Discussion

l‐homoarginine has a well‐established role to alter endothelial function in cardiovascular diseases, yet its role in diabetic kidney injury has not previously been determined. This study shows that homoarginine supplementation mediates renal tissue protection as demonstrated by a reduction in albuminuria, histopathological changes, and kidney macrophage recruitment during diabetes. We speculate that the effect of homoarginine in the kidney is mainly mediated via increased NO production as indicated by increased kidney nitrate + nitrite levels in diabetic mice. These findings reveal an important role for homoarginine in the pathogenesis of DN and provide evidence for homoarginine supplementation as potential therapeutic modality for treating diabetic patients.

Although l‐homoarginine has been known for a century, its role in physiology and pathophysiology remains unclear. l‐homoarginine is synthesized by mitochondrial AGAT in kidneys using l‐arginine and l‐lysine as substrates (Ryan and Wells 1964; Choe et al. 2013). Clinical studies of l‐arginine metabolites indicated that reduced l‐homoarginine levels were correlated with coronary heart diseases, peripheral arterial occlusive disease, stroke, and overall mortality (Marz et al. 2010; Ravani et al. 2013; Drechsler et al. 2015; Pilz et al. 2015). Choe et al. (2013) reported that elevated l‐homoarginine levels were associated with reduced all‐cause mortality in patients with ischemic stroke and that low l‐homoarginine appears to be related to poor outcome after ischemic stroke. Moreover, l‐homoarginine supplementation significantly improved cerebral damage and neurological deficits in mouse models with stroke (Haghikia et al. 2017).

Clinical investigations demonstrated that l‐homoarginine concentrations are reduced in patients with impaired kidney function. The European mild to moderate kidney disease study showed that plasma l‐homoarginine levels were significantly associated with reduced glomerular filtration rate (GFR) and proteinuria (Ravani et al. 2013), and low plasma l‐homoarginine concentrations were identified as a predictor of kidney progression (Drechsler et al. 2013; Ravani et al. 2013). Importantly, l‐homoarginine supplementation has been shown to be safe and well tolerated in young volunteers (Atzler et al. 2016). In addition, supplementation of homoarginine to control mice did not alter any hemodynamic parameters or body weight despite increasing plasma homoarginine levels (Atzler et al. 2017; Karetnikova et al. 2019). Although reduced l‐homoarginine levels are a marker for cardiovascular and kidney disease (Morris et al. 2011; Jud et al. 2018; Martens‐Lobenhoffer et al. 2018), the role of l‐homoarginine in diabetes remains controversial (Carmann et al. 2015; Krebs et al. 2015). In a diet‐induced obese mouse model, Stockebrand et al. (2015) reported that l‐homoarginine supplementation reduced blood glucose and stimulated insulin production, despite having no effect on body weight or glucose tolerance. Although we found reduced plasma l‐homoarginine levels in diabetic *Ins2^Akita^* mice, our data showed no effect of l‐homoarginine supplementation on elevated blood glucose levels in diabetic *Ins2^Akita^* mice. However, l‐homoarginine supplementation did attenuate diabetes‐induced reductions in albuminuria, kidney histological changes, glomerular macrophage recruitment, KC‐GRO/CXCL1 levels, urinary TBARS excretion, and restored kidney nitrate + nitrite levels.

Although diabetes significantly reduced l‐arginine levels in plasma and kidney tissues as reported previously (Morris et al. 2011; You et al. 2013b, 2014), l‐homoarginine supplementation did not affect these parameters in our current study, indicating that the renal protective effect of l‐homoarginine is likely unrelated to reduced l‐arginine availability. However, our results indicate possible direct or indirect effects of l‐homoarginine on NO production. This notion is derived from our data showing restoration of kidney nitrate + nitrite levels and reduced urinary TBARS in diabetic mice. The effects of homoarginine on NO synthesis have been linked with increased risk of stroke and atherosclerosis (Haghikia et al. 2017). DN is associated with endothelial nitric oxide synthase (eNOS) uncoupling, increased reactive oxygen species (ROS), and oxidative stress in diabetic kidneys (Faria et al. 2012). The fact that l‐homoarginine is a weak substrate for NOS (Hecker et al. 1991; Moali et al. 1998) raises the possibility that it may directly affect NO production. Alternatively, the effects of l‐homoarginine on kidney nitrate + nitrite may be mediated indirectly, for example, via changes in levels of asymmetric dimethylarginine (ADMA), an endogenous inhibitor of NOS. The basis for the possible effects of l‐homoarginine on NO production in DN is not known and will be investigated in future studies.

In summary, this study demonstrates that l‐homoarginine is reduced in diabetic mice and that l‐homoarginine supplementation protects against specific features of renal damage in diabetes, thus suggesting that l‐homoarginine supplementation may be useful in treatment of DN. Future research will determine the specific mechanisms by which l‐homoarginine affects DN and whether l‐homoarginine administration alters specific aspects of arginine metabolism that are altered in diabetes.

## Conflict of Interest

All authors declare no conflict of interest.

## References

[phy214235-bib-0001] Atzler, D. , M. Rosenberg , M. Anderssohn , C. U. Choe , M. Lutz , C. Zugck , et al. 2013 Homoarginine–an independent marker of mortality in heart failure. Int. J. Cardiol. 168:4907–4909. 23890923 10.1016/j.ijcard.2013.07.099

[phy214235-bib-0002] Atzler, D. , M. O. Gore , C. R. Ayers , C. U. Choe , R. H. Boger , J. A. de Lemos , et al. 2014a Homoarginine and cardiovascular outcome in the population‐based Dallas Heart Study. Arterioscler. Thromb. Vasc. Biol. 34:2501–2507. 25189571 10.1161/ATVBAHA.114.304398

[phy214235-bib-0003] Atzler, D. , E. Schwedhelm , M. Nauck , T. Ittermann , R. H. Boger , and N. Friedrich . 2014b Serum reference intervals of homoarginine, ADMA, and SDMA in the study of health in Pomerania. Clin. Chem. Lab. Med. 52:1835–1842. 24945429 10.1515/cclm-2014-0314

[phy214235-bib-0004] Atzler, D. , M. Schonhoff , K. Cordts , I. Ortland , J. Hoppe , F. C. Hummel , et al. 2016 Oral supplementation with l‐homoarginine in young volunteers. Br. J. Clin. Pharmacol. 82:1477–1485. 27434056 10.1111/bcp.13068 PMC5099544

[phy214235-bib-0005] Atzler, D. , D. J. McAndrew , K. Cordts , J. E. Schneider , S. Zervou , E. Schwedhelm , et al. 2017 Dietary supplementation with homoarginine preserves cardiac function in a murine model of post‐myocardial infarction heart failure. Circulation 135:400–402. 28115416 10.1161/CIRCULATIONAHA.116.025673

[phy214235-bib-0006] Awad, A. S. , G. R. Kinsey , K. Khutsishvili , T. Gao , W. K. Bolton , and M. D. Okusa . 2011 Monocyte/macrophage chemokine receptor CCR6 mediates diabetic renal injury. Am. J. Physiol. Renal Physiol. 301:F1358–F1366. 21880831 10.1152/ajprenal.00332.2011 PMC3233863

[phy214235-bib-0007] Awad, A. S. , H. You , T. Gao , T. K. Cooper , S. A. Nedospasov , J. Vacher , et al. 2015a Macrophage‐derived tumor necrosis factor‐α mediates diabetic renal injury. Kidney Int. 88:722–733. 26061548 10.1038/ki.2015.162 PMC4589442

[phy214235-bib-0008] Awad, A. S. , H. You , T. Gao , A. Gvritishvili , T. K. Cooper , and J. Tombran‐Tink . 2015b Delayed treatment with a small pigment epithelium derived factor (PEDF) peptide prevents the progression of diabetic renal injury. PLoS ONE 10:e0133777. 26207369 10.1371/journal.pone.0133777 PMC4514848

[phy214235-bib-0009] Bahls, M. , D. Atzler , M. R. P. Markus , N. Friedrich , R. H. Boger , H. Volzke , et al. 2018 Low‐Circulating Homoarginine is associated with dilatation and decreased function of the left ventricle in the general population. Biomolecules 8:63. 10.3390/biom8030063 PMC6165018 30061520

[phy214235-bib-0010] Boyle, J. P. , T. J. Thompson , E. W. Gregg , L. E. Barker , and D. F. Williamson . 2010 Projection of the year 2050 burden of diabetes in the US adult population: dynamic modeling of incidence, mortality, and prediabetes prevalence. Popul Health Metr 8:29. 20969750 10.1186/1478-7954-8-29 PMC2984379

[phy214235-bib-0011] Breyer, M. D. , E. Bottinger , F. C. 3rd Brosius , T. M. Coffman , R. C. Harris , C. W. Heilig , et al. 2005 Mouse models of diabetic nephropathy. J. Am. Soc. Nephrol. 16:27–45. 15563560 10.1681/ASN.2004080648

[phy214235-bib-0012] Brosius, F. C. 3rd , C. E. Alpers , E. P. Bottinger , M. D. Breyer , T. M. Coffman , S. B. Gurley , et al. 2009 Mouse models of diabetic nephropathy. J. Am. Soc. Nephrol. 20:2503–2512. 19729434 10.1681/ASN.2009070721 PMC4075053

[phy214235-bib-0013] Carmann, C. , E. Lilienthal , K. Weigt‐Usinger , A. Schmidt‐Choudhury , I. Horster , A. A. Kayacelebi , et al. 2015 The l‐arginine/NO pathway, homoarginine, and nitrite‐dependent renal carbonic anhydrase activity in young people with type 1 diabetes mellitus. Amino Acids 47:1865–1874. 26123986 10.1007/s00726-015-2027-9

[phy214235-bib-0014] Choe, C. U. , D. Atzler , P. S. Wild , A. M. Carter , R. H. Boger , F. Ojeda , et al. 2013 Homoarginine levels are regulated by l‐arginine:glycine amidinotransferase and affect stroke outcome: results from human and murine studies. Circulation 128:1451–1461. 24004504 10.1161/CIRCULATIONAHA.112.000580

[phy214235-bib-0015] Citro, A. , E. Cantarelli , and L. Piemonti . 2015 The CXCR15/2 pathway: involvement in diabetes pathophysiology and potential target for T1D interventions. Curr. Diab. Rep. 15:68. 26275440 10.1007/s11892-015-0638-x

[phy214235-bib-0016] Drechsler, C. , B. Kollerits , A. Meinitzer , W. Marz , E. Ritz , P. Konig , et al. 2013 Homoarginine and progression of chronic kidney disease: results from the Mild to Moderate Kidney Disease Study. PLoS ONE 8:e63560. 23691067 10.1371/journal.pone.0063560 PMC3655120

[phy214235-bib-0017] Drechsler, C. , H. Pihlstrom , A. Meinitzer , S. Pilz , A. Tomaschitz , S. Abedini , et al. 2015 Homoarginine and clinical outcomes in renal transplant recipients: results from the assessment of lescol in renal transplantation study. Transplantation 99:1470–1476. 25675199 10.1097/TP.0000000000000568

[phy214235-bib-0018] Faria, A. M. , A. Papadimitriou , K. C. Silva , J. M. Lopes de Faria , and J. B. Lopes de Faria . 2012 Uncoupling endothelial nitric oxide synthase is ameliorated by green tea in experimental diabetes by re‐establishing tetrahydrobiopterin levels. Diabetes 61:1838–1847. 22586583 10.2337/db11-1241 PMC3379677

[phy214235-bib-0019] Haghikia, A. , G. R. Yanchev , A. A. Kayacelebi , E. Hanff , N. Bledau , C. Widera , et al. 2017 The role of l‐arginine/l‐homoarginine/nitric oxide pathway for aortic distensibility and intima‐media thickness in stroke patients. Amino Acids 49:1111–1121. 28285332 10.1007/s00726-017-2409-2

[phy214235-bib-0020] Hecker, M. , D. T. Walsh , and J. R. Vane . 1991 On the substrate specificity of nitric oxide synthase. FEBS Lett. 294:221–224. 1721880 10.1016/0014-5793(91)81434-a

[phy214235-bib-0021] Jud, P. , F. Hafner , N. Verheyen , A. Meinitzer , T. Gary , M. Brodmann , et al. 2018 Homoarginine/ADMA ratio and homoarginine/SDMA ratio as independent predictors of cardiovascular mortality and cardiovascular events in lower extremity arterial disease. Sci. Rep. 8:14197. 30242192 10.1038/s41598-018-32607-8 PMC6155043

[phy214235-bib-0022] Karetnikova, E. S. , N. Jarzebska , A. G. Markov , N. Weiss , S. R. Lentz , and R. N. Rodionov . 2019 Is homoarginine a protective cardiovascular risk factor? Arterioscler. Thromb. Vasc. Biol. 39:869–875. ATVBAHA118312218. 30866658 10.1161/ATVBAHA.118.312218

[phy214235-bib-0023] Krebs, A. , J. Doerfer , S. C. Grunert , J. Wohrl , B. Stier , A. Schmidt‐Trucksass , et al. 2015 Decreased levels of homoarginine and asymmetric dimethylarginine in children with type 1 diabetes: associations with cardiovascular risk factors but no effect by atorvastin. J. Pediatr. Endocrinol. Metab. 28:147–152. 25153574 10.1515/jpem-2014-0083

[phy214235-bib-0024] Martens‐Lobenhoffer, J. , I. E. Emrich , A. M. Zawada , D. Fliser , S. Wagenpfeil , G. H. Heine , et al. 2018 l‐Homoarginine and its AGXT2‐metabolite GOCA in chronic kidney disease as markers for clinical status and prognosis. Amino Acids 50:1347–1356. 29982953 10.1007/s00726-018-2610-y

[phy214235-bib-0025] Marz, W. , A. Meinitzer , C. Drechsler , S. Pilz , V. Krane , M. E. Kleber , et al. 2010 Homoarginine, cardiovascular risk, and mortality. Circulation 122:967–975. 20733103 10.1161/CIRCULATIONAHA.109.908988

[phy214235-bib-0026] Moali, C. , J. L. Boucher , M. A. Sari , D. J. Stuehr , and D. Mansuy . 1998 Substrate specificity of NO synthases: detailed comparison of l‐arginine, homo‐l‐arginine, their N omega‐hydroxy derivatives, and N omega‐hydroxynor‐l‐arginine. Biochemistry 37:10453–10460. 9671515 10.1021/bi980742t

[phy214235-bib-0027] Morris, S. M. Jr , T. Gao , T. K. Cooper , D. Kepka‐Lenhart , and A. S. Awad . 2011 Arginase‐2 mediates diabetic renal injury. Diabetes 60:3015–3022. 21926276 10.2337/db11-0901 PMC3198072

[phy214235-bib-0028] Morris, S. M. Jr , H. You , T. Gao , J. Vacher , T. K. Cooper , and A. S. Awad . 2017 Distinct roles of arginases 1 and 2 in diabetic nephropathy. Am. J. Physiol. Renal Physiol. 313:F899–F905. 28446459 10.1152/ajprenal.00158.2017 PMC5668588

[phy214235-bib-0029] Pilz, S. , F. Edelmann , A. Meinitzer , G. Gelbrich , U. Doner , H. D. Dungen , et al. 2014a Associations of methylarginines and homoarginine with diastolic dysfunction and cardiovascular risk factors in patients with preserved left ventricular ejection fraction. J. Card. Fail. 20:923–930. 25230239 10.1016/j.cardfail.2014.09.004

[phy214235-bib-0030] Pilz, S. , T. Teerlink , P. G. Scheffer , A. Meinitzer , F. Rutters , A. Tomaschitz , et al. 2014b Homoarginine and mortality in an older population: the Hoorn study. Eur. J. Clin. Invest. 44:200–208. 24251815 10.1111/eci.12208

[phy214235-bib-0031] Pilz, S. , A. Meinitzer , M. Gaksch , M. Grubler , N. Verheyen , C. Drechsler , et al. 2015 Homoarginine in the renal and cardiovascular systems. Amino Acids 47:1703–1713. 25929587 10.1007/s00726-015-1993-2

[phy214235-bib-0032] Ravani, P. , R. Maas , F. Malberti , P. Pecchini , M. Mieth , R. Quinn , et al. 2013 Homoarginine and mortality in pre‐dialysis chronic kidney disease (CKD) patients. PLoS ONE 8:e72694. 24023762 10.1371/journal.pone.0072694 PMC3762798

[phy214235-bib-0033] Ryan, W. L. , and I. C. Wells . 1964 Homocitrulline and homoarginine synthesis from lysine. Science 144:1122–1127. 14148430 10.1126/science.144.3622.1122

[phy214235-bib-0034] Sobczak, A. , A. Prokopowicz , M. Radek , M. Szula , M. Zaciera , J. Kurek , et al. 2014 Tobacco smoking decreases plasma concentration of the emerging cardiovascular risk marker, l‐homoarginine. Circ. J. 78:1254–1258. 24583919 10.1253/circj.cj-13-1334

[phy214235-bib-0035] Stockebrand, M. , S. Hornig , A. Neu , D. Atzler , K. Cordts , R. H. Boger , et al. 2015 Homoarginine supplementation improves blood glucose in diet‐induced obese mice. Amino Acids 47:1921–1929. 26077714 10.1007/s00726-015-2022-1

[phy214235-bib-0036] Valtonen, P. , T. Laitinen , T. Lyyra‐Laitinen , O. T. Raitakari , M. Juonala , J. S. Viikari , et al. 2008 Serum l‐homoarginine concentration is elevated during normal pregnancy and is related to flow‐mediated vasodilatation. Circ. J. 72:1879–1884. 18802314 10.1253/circj.cj-08-0240

[phy214235-bib-0037] Vogl, L. , J. Pohlhammer , A. Meinitzer , B. Rantner , M. Stadler , S. Peric , et al. 2015 Serum concentrations of l‐arginine and l‐homoarginine in male patients with intermittent claudication: a cross‐sectional and prospective investigation in the CAVASIC Study. Atherosclerosis 239:607–614. 25746168 10.1016/j.atherosclerosis.2015.02.019

[phy214235-bib-0038] Wang, C. H. , F. Li , S. Hiller , H. S. Kim , N. Maeda , O. Smithies , et al. 2011 A modest decrease in endothelial NOS in mice comparable to that associated with human NOS3 variants exacerbates diabetic nephropathy. Proc. Natl. Acad. Sci. USA 108:2070–2075. 21245338 10.1073/pnas.1018766108 PMC3033253

[phy214235-bib-0039] You, H. , T. Gao , T. K. Cooper , W. Brian Reeves , and A. S. Awad . 2013a Macrophages directly mediate diabetic renal injury. Am. J. Physiol. Renal Physiol. 305:F1719–F1727. 24173355 10.1152/ajprenal.00141.2013 PMC3882451

[phy214235-bib-0040] You, H. , T. Gao , T. K. Cooper , S. M. Jr Morris , and A. S. Awad . 2013b Arginase inhibition mediates renal tissue protection in diabetic nephropathy by a nitric oxide synthase 3‐dependent mechanism. Kidney Int. 84:1189–1197. 23760286 10.1038/ki.2013.215 PMC3783645

[phy214235-bib-0041] You, H. , T. Gao , T. K. Cooper , S. M. Jr Morris , and A. S. Awad . 2014 Diabetic nephropathy is resistant to oral l‐arginine or l‐citrulline supplementation. Am. J. Physiol. Renal Physiol. 307:F1292–F1301. 25320354 10.1152/ajprenal.00176.2014 PMC4254967

[phy214235-bib-0042] You, H. , T. Gao , T. K. Cooper , S. M. Morris , and A. S. Awad . 2015 Arginase inhibition: a new treatment for preventing progression of established diabetic nephropathy. Am. J. Physiol. Renal Physiol. 309:F447–F455. 26041444 10.1152/ajprenal.00137.2015 PMC4556892

[phy214235-bib-0043] You, H. , T. Gao , W. M. Raup‐Konsavage , T. K. Cooper , S. K. Bronson , W. B. Reeves , et al. 2017 Podocyte‐specific chemokine (C‐C motif) receptor 2 overexpression mediates diabetic renal injury in mice. Kidney Int. 91:671–682. 27914709 10.1016/j.kint.2016.09.042 PMC5313320

[phy214235-bib-0044] Zhao, H. J. , S. Wang , H. Cheng , M. Z. Zhang , T. Takahashi , A. B. Fogo , et al. 2006 Endothelial nitric oxide synthase deficiency produces accelerated nephropathy in diabetic mice. J. Am. Soc. Nephrol. 17:2664–2669. 16971655 10.1681/ASN.2006070798 PMC4618687

[phy214235-bib-0045] van der Zwan, L. P. , M. Davids , P. G. Scheffer , J. M. Dekker , C. D. Stehouwer , and T. Teerlink . 2013 l‐Homoarginine and l‐arginine are antagonistically related to blood pressure in an elderly population: the Hoorn study. J. Hypertens. 31:1114–1123. 23552121 10.1097/HJH.0b013e32836037fb

